# Bioavailability and bioaccumulation characterization of essential and heavy metals contents in *R. acetosa, S. oleracea and U. dioica* from copper polluted and referent areas

**DOI:** 10.1186/s40201-015-0159-1

**Published:** 2015-01-24

**Authors:** Biljana Balabanova, Trajče Stafilov, Katerina Bačeva

**Affiliations:** Faculty of Agriculture, Goce Delčev University, Krste Misirkov bb, 2000 Štip, Macedonia; Institute of Chemistry, Faculty of Science, Ss. Cyril and Methodius University, Arhimedova 5, 1000 Skopje, Macedonia; Research Center for Environment and Materials, Macedonian Academy of Sciences and Arts, Krste Misirkov 2, 1000 Skopje, Republic of Macedonia

**Keywords:** Metals, Bioaccumulation, *Rumex acetosa*, *Spinacia oleracea*, *Urtica dioica*, ICP-AES

## Abstract

**Background:**

Bioavailability of metals occurring in soil is the basic source of its accumulation in vegetables and herbs. The impact of soil pollution (due to urban and mining areas) on the food chain presents a challenge for many investigations. Availability of metals in a potentially polluted soil and their possible transfer and bioaccumulation in sorrel (*Rumex acetosa*), spinach (*Spinacia oleracea*) and common nettle (*Urtica dioica*), were examined.

**Methods:**

Microwave digestion was applied for total digestion of the plant tissues, while on the soil samples open wet digestion with a mixture of acids was applied. Three extraction methods were implemented for the bioavailable metals in the soil. Atomic emission spectrometry with inductively coupled plasma was used for determination of the total contents of 21 elements.

**Results:**

Significant enrichments in agricultural soil for As, Pb and Zn (in urban area), Cd, Cu and Ni (in a copper mine area), compared with the respective values from European standards were detected. On the basis of three different extraction methods, higher availability was assumed for both lithogenic and anthropogenic elements. Translocation values >1 were obtained for As, Cd, Cu, Ni, Pb and Zn. Higher bioconcentrating value was obtained only for Cd, while the bioaccumulation values vary from 0.17 for Cd to 0.82 for Zn.

**Conclusions:**

The potential availability of hazardous metals in urban and mining soils is examined using DTPA-TEA-CaCl_2_ (urban) and HCl (Cu-mines areas). Our results suggested that *S. oleracea* and *R. acetosa* have a phytostabilization potential for Cd, Cu, Ni and Pb, while *U. dioica* only for Cu. *R. acetosa* has a potential for phytoextraction of Cd in urban and copper polluted areas.

## Background

Since food contamination is one of the major routes for entry of metals into the humans and animals, monitoring the bioavailability pools of metals in contaminated vegetables has generated a great interest [1-5]. Pertaining to this awareness, a lot of studies have been conducted for determining the metals contents in the vegetables and herbs that are included in human diet [6-8]. Vegetables/herbs contamination with heavy metals derives primarily from contaminated soil [4]. The uptake of metals from the soil depends on different factors such as their soluble content, soil pH, plant growth stages, types of species, etc. [9-11]. Plants have developed different strategies to grow on agricultural soils rich in heavy metals [1,4,5]. In this way, the food presents the main source of human exposure to toxic metals [12,3]. Different plant parts accumulate different levels of these metals. For example, higher metal concentrations were observed in the edible and inedible parts of vegetable species as reported by many authors [13-15]. Considering this, sorrel (*Rumex acetosa L*.), spinach (*Spinacia oleracea*) and common nettle (*Urtica dioica L*.) were used as vegetable/herb samples for investigating the possible toxic metals bioaccumulation and transferring ability from reference and polluted agricultural lands (mines and urban areas). Appropriately, mines’ surroundings and urban areas were selected as investigation areas to determine the anthropogenic impact on the agricultural land and food quality (in terms of pollution with toxic metals). Previous investigation shows that the “Bučim” copper mine and the former “Damjan” iron mine have a significant impact on air and soil pollution in their close environs [16-19]. However, the environmental impact on the food chain has been very poorly investigated. Most frequent leaf vegetables and herbs in the investigated area were sorrel, spinach and common nettle. The sorrel and spinach as vegetables, and the common nettle as herb and food additive, are commonly used in the local population’s diet and globally, they are numbered among the most popular foods for their high content of vitamins and minerals [20].

The main goal of this work is to assess the complex phenomenon of essential and toxic metals distribution in the food chain in the old mining areas. As main markers of pollution in agricultural land and vegetables two types of markers were used: simple markers, represented by the elements contents in the contaminated soils and by the elements contents in the vegetables consumed by the population in the polluted and unpolluted areas. Sorrel (*Rumex acetosa L*.), spinach (*Spinacia oleracea*) and common nettle (*Urtica dioica L*.) have been cultivated in the contaminated agricultural land of the local settlements for a long period of time now, and this is why their phytostabilization and phytoextraction ability were also examined.

## Methods

### Sampling and sampling site characterization

**Figure 1:**
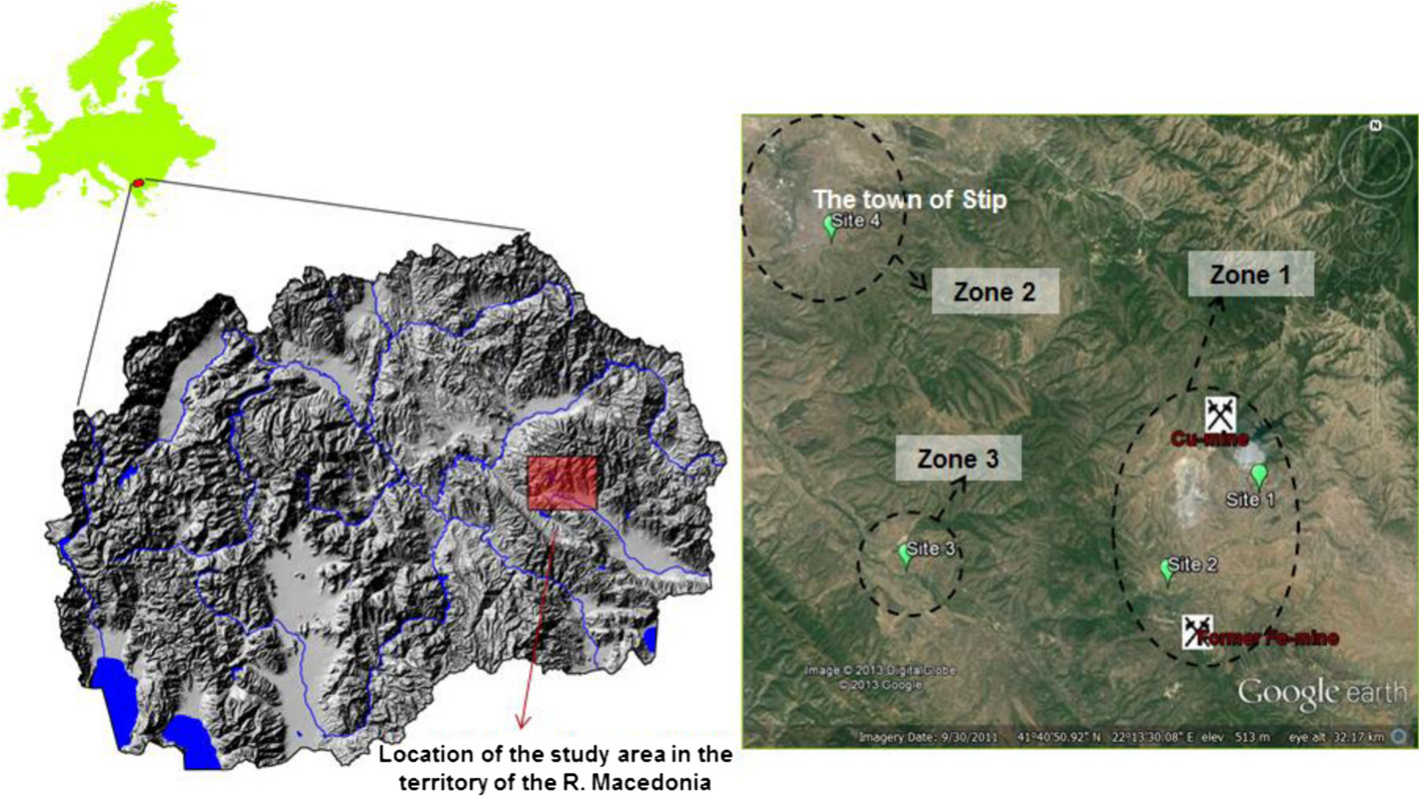
**Locality of the study area on the territory of the R. Macedonia divided in zones.** Zone 1 – polluted area; Zone 2 – urban area; Zone 3 – control area, non-contaminated area.

Plant species and soil samples were collected from four localities (Figure 1, Table 1). The first locality (site 1) - village Topolnica was the most polluted region, affected by the activities of “Bučim” copper mine and flotation tailings [16-18]. The second locality (site 2) is village Damjan, air-distanced 4 km from the flotation tailings dam and 2 km from the ore wastes dam of “Bučim” copper mine. This settlement is also affected by the former iron mine “Damjan”, as given by Balabanova et al. [18]. The third sampling location (site 3), village Lakavica, was 12 km distanced from the pollution source. Previous investigation of air distribution (using plant species moss and lichens) determined no significant distribution of air transported metals from the copper mine. Therefore, this area can be used as a reference area. The fourth sampling locality (site 4), the town of Štip, was used for measuring the impact in the urban environment. A total of 10 plant samples including roots, stems and leaves of each species, were collected from each site, mixed together to form a composite sample, and placed in labeled bags for further analysis. Soil samples (5 replicates), at 0–20 cm depth from the rhizosphere of each plant, were taken from each site where from the plant sample was found. Soil samples (a composite mixture) were air dried at room temperature for two weeks, crushed and pulverized to pass through a 2-mm sieve.

**Table 1:** **Geographical coordinates of sampling locations**

**Site**	**Locality**	**Site characterization**	**E**	**N**
**1**	Village Topolnica	Cu-mine environ	22°22′47.00″	41°39′41.73″
**2**	Village Damjan	Former Fe mine environ	22°20′33.91″	41°37′57.03″
**3**	Village Lakavica	Reference area	22°14′7.95″	41°38′13.91″
**4**	Town of Štip	Urban area	22°12′16.23″	41°44′16.90″

### Sample pre-treatment for elements contents analysis

#### Soil digestion (total and extractability forms)

For total digestion, an open wet digestion method with a mixture of acids was applied. Precisely measured masses of soil samples (0.2500 g) were placed in a Teflon digestion vessel and were digested on a hot plate at temperature 150-180°C. At first, HNO_3_ was added to remove all organic matters, then a mixture of HF and HClO_4_ was added, followed by a third step where HCl and water were added to dissolve the residues. The solution was quantitatively transferred to a 25 mL volumetric flask [18].

Three methods were applied for the study of the plant-availability of the elements: (1) Extraction with deionized H_2_O that provides information on the actual availability of the elements in the soil solution; (2) Extraction with 0.1 M HCl for 1 h and filtering through an acid-resistant filter, displacing potentially available forms that are not easily extracted. The immobilization of the heavy metals in the contaminated soil is carried out through chelating and washing with mineral acids. In this way the efficiency in the extraction of heavy metals from the soil is determined. An effective pH level is required to enable remediation of heavy metals from contaminated soils. Because of the differences in the affinities of heavy metals with soil and soil constituents the effective pH levels are likely to vary depending on the type of the heavy metals and the strength of their bonding with the soil. (3) Extraction of the soluble species of trace elements in a mixed buffered solution (pH = 7.3) of triethanolamine (TEA, 0.1 mol L^−1^) with CaCl_2_ (0.01 mol L^−1^) and diethylenetriaminepentaacetic acid (DTPA, 0.005 mol L^−1^), which is often recommended for extraction of toxic or biogenic metals. The DTPA extracting solution was prepared in the following way: 0.005 mol L^−1^ DTPA, 0.01 mol L^−1^ CaCl_2_ and 0.1 mol L^−1^ TEA was adjusted to pH to 7.30 ± 0.05 with 1:1 HCl [21].

#### Plants digestion

The plants collected from the agricultural soils were washed with water and rinsed with distillated water, to ensure that they are thoroughly cleaned and any outside contamination has been removed. Every plant species was separated root from shoot. Plant samples were dried at room temperature for almost two weeks, pulverized and passed through a 2 mm stainless steel sieve. In this way representative samples were prepared.

For digestion of the plant tissue samples, a microwave digestion system (CEM, model Mars, USA) was applied. 0.5 g of plant tissue samples were precisely (with accuracy of 0.0001) measured, then 5 mL concentrated nitric acid, HNO_3_ (69%, 108 *m/V*, trace pure) and 2 mL hydrogen peroxide, H_2_O_2_ (30%, *m/V*, trace pure) were added. The Teflon vessels were carefully closed and a microwave digestion method was applied. The digestion method was performed in two steps for total dissolution of the plants tissue at 180°C and pressure of 600 psi applying 100% of 1600 W power energy [16,17,21]. When the digestion was completed, the digests were quantitatively transferred into 25 mL volumetric flaks. The digests from plant tissue prepared in this way were then analysed for the total elements contents.

#### Spectroscopic analysis

The total contents of 21 elements were analyzed: (1) macro biogenic elements (Ca, K, Na, Mg, and P), (2) elements that have the essential functionality in micro-contents (Ba, Cr, Li, Cu, Fe, Mo, Mn, Sr and Zn), and elements that are toxic even in traces (Ag, Al, As, Cd, Ni, Pb and V). The elements contents were determined using atomic emission spectrometry with inductively coupled plasma, ICP-AES (Varian, model 715-ES, USA) with application of an ultrasonic nebuliser CETAC (ICP/U-5000AT+) for improved sensitivity for the elements contents in the plants digest.

In addition, quality control was assured by standard reference materials (moss as plant species), M2 and M3 moss plant tissue (for the plant species) and JSAC 0401 as soil reference material. The difference between measured and certified values for all analyzed elements was satisfactory, ranging within 10%. The internal standard addition method was applied to all measured elements, and the recoveries of all elements were in the range of 96.6-103%. The method sensitivity in regard to the lower limit of detection of the elements contents in the plants digest was improved using an ultrasonic nebuliser: 0.001 mg kg^−1^ for Mn and Li; 0.0125 mg kg^−1^ for Al, Cu; 0.002 mg kg^−1^ for Fe; 0.003 mg kg^−1^ for Zn; 0.005 mg kg^−1^ for Cd; 0.01 mg kg^−1^ for Ag, Mo, V; 0.025 mg kg^−1^ for Ba, Ca, Mg, Sr; 0.05 mg kg^−1^ for Cr; 0.25 mg kg^−1^ for Ni; 0.25 mg kg^−1^ for As, P, Pb; 2.5 mg kg^−1^ for Na; 5 mg kg^−1^ for K. The values for the soil digest are maximized for 10% using a standard nebuliser.

#### Data processing

The values obtained for the contents of the investigated elements were statistically processed using basic descriptive statistics. Box–Cox transformation was used for data transformation [22,23]. Bivariate statistic method was applied to check the data about the correlations between elements contents. Multivariate statistic methods (principal component analysis-PCA and factor analysis-FA) were used to reveal the associations of the chemical elements [24-26]. Biological Concentration Factor (BCF) was calculated as metal concentration ratio of plant roots to soil as given by Malik et al. [3] and Yoon et al. [6]. Translocation Factor (TF) was described as ratio of heavy metals in plant shoot to that in plant root [7,27]. Biological Accumulation Factor (BAF) was calculated as ratio of heavy metal in shoots to that in the soil [3,7,27].

## Results and discussion

### Soil analysis

#### Characterization of metals contents in soils

The total contents of the analyzed elements in the soil were analyzed in order to characterize possible soil pollution and to determine the transfer efficiency from the soil. The distribution of the macro elements varies among the localities in the investigated area, yet the median values are in line with certain European standards [28]. The maximum contents for Ca and P were obtained in the agricultural land from the urban site (3.5 and 0.2%, respectively, Table 2). The maximum contents for K and Mg were obtained from the agricultural land in the Cu-mine environ (2.3 and 1.1%, respectively). This occurrence is due to the intensive use of organic fertilizers on the rural agricultural land. The most enriched Al content was obtained in the Fe-mine environs, due to geochemistry of the soil minerals [18]. The median values for the total contents of As, Cd, Cr, Cu, Fe, Ni, Pb, and Zn in the soil showed that these elements are found in higher contents compared to the European average values (Table 2). Thus, the European mean values for As, Cd, Cr, Cu, Fe, Ni, Pb and Zn are 11.6, 0.28, 94.8, 17.3, 38 000, 21.8, 32.6, 68.1 mg kg^−1^ respectively, as given by Salminen et al. [28]. Comparing these values with the respective values obtained in this study, enrichment ratios (ER) were calculated: ER_As_ = 2.5, ER_Cd_ = 3.1, ER_Cu_ = 4.01, ER_Ni_ = 1.33, ER_Pb_ = 1.46, ER_Zn_ = 1.48. It can be noticed that there are no significant enrichment considering the Cr and Fe contents [28]. However, the total Cr contents quantified in the agricultural land can be considered as not contaminated. The iron contents shows enrichment only in the agricultural land in the Cu-mine environ, ER_Fe_ = 1.13, compared to the European average for Fe contents in soil [28]. Despite the detected enrichments, the respective level of its content in the soil is not considered as toxic to the plants, according to Marschner [11]. Considering this, it can be concluded that the analyzed agriculture soils are polluted with As, Cd, Cu and Pb. The maximum values for Cd (1.06 mg kg^−1^) and Cu (100 mg kg^−1^) were obtained from the soil samples from the very close surroundings of the copper mine (marked as bold values in Table 2). The maximum values for As and Pb were obtained from the urban area soil samples (49 and 84 mg kg^−1^, respectively). In the same agricultural land the nutritive mineral Zn contents reaches to 181 mg kgkg^−1^ (Table 2). For almost all analyzed elements no significant variations were detected between the total contents in the soil sampled from site 2 and site 3.

**Table 2:** **The contents of the analyzed elements in soil (total and extractability contents), given in mg kg** ^**−1**^ **, n = 4**

**Element**	**Extraction in H**_**2**_ **O**	**Extraction in 0.1 mol L** ^**−1**^ **HCl**	**Extraction in DTPA–CaCl**_**2**_ **–TEA**	**Total contents**			
				**Copper mine environ (site 1)**	**Former iron mine environ (site 2)**	**Reference area (site 3)**	**Urban area (site 4)**
**Ag**	<0.01	0.02 ± 0.01	<0.01	0.47	0.76	0.46	1.09
**Al**	23.9 ± 16.5	**62.9 ± 46.2**	0.78 ± 0.88	58992	61061	59467	59643
**As**	<0.25	**<0.25**	<0.25	**26.9**	23.4	18.9	**48.7**
**Ba**	0.18 ± 0.05	**10.9 ± 5.81**	0.71 ± 0.40	311	403	343	437
**Ca**	104 ± 47.7	**3421 ± 430**	1222 ± 197	22677	20447	21954	35394
**Cd**	0.01 ± 0.001	0.05 ± 0.03	0.05 ± 0.03	**1.06**	0.96	0.86	0.59
**Cr**	0.04 ± 0.02	0.06 ± 0.03	0.01 ± 0.003	**63.6**	41.9	55.8	54.2
**Cu**	0.30 ± 0.06	1.57 ± 1.97	**8.60 ± 6.65**	**100**	63.7	34.3	79.0
**Fe**	27.9 ± 18.1	4.43 ± 4.84	28.0 ± 33.4	**43025**	32629	32646	31242
**K**	56.2 ± 15.6	**155 ± 47.2**	81.6 ± 26.6	23716	19805	16173	20290
**Li**	0.01 ± 0.004	0.04 ± 0.006	0.006 ± 0.005	7.79	5.70	7.74	9.81
**Mg**	21.7 ± 6.15	**305 ± 79.6**	103 ± 28.7	10676	6583	8956	8629
**Mn**	0.47 ± 0.31	**44.4 ± 28.5**	10.6 ± 2.82	521	577	575	567
**Mo**	0.04 ± 0.04	0.04 ± 0.02	0.03 ± 0.03	4.0	3.2	2.8	2.5
**Na**	6.91 ± 4.67	9.73 ± 5.94	6.06 ± 5.35	8582	**9248**	8506	7956
**Ni**	0.18 ± 0.05	**0.84 ± 0.54**	0.55 ± 0.22	19.6	**39.0**	23.9	34.0
**P**	16.6 ± 3.75	**177 ± 24.8**	5.19 ± 1.41	1385	931	995	**2184**
**Pb**	0.25 ± 0.07	0.38 ± 0.26	**1.71 ± 1.15**	24.4	44.8	36.8	**83.9**
**V**	0.08 ± 0.03	0.10 ± 0.08	0.06 ± 0.04	**93.8**	72.2	82.7	67.9
**Zn**	0.28 ± 0.11	**6.02 ± 5.34**	5.02 ± 5.10	78.8	72.9	73.2	**181**

Bold values indicated the maximum extracted content of current element under specific extraction reagents.

Different levels of availability can be estimated depending on the extracting power of the extraction reagent that was used. Very low variations were identified between the extraction solutions for Cd, Cr and Ni. It was found that 1.6% of arsenic were extracted using 0.1 mol L^−1^ HCl ranging from 0.07 mg kg^−1^ in the reference area (Site 3) to 0.35 mg kg^−1^ in the urban area agricultural soil- site 4 (Table 2). The sequestering reagent was much more effective in the case of Cu and Pb (extracted contents of 12.4% and 3.6%, respectively) as presented in Table 2. Despite the median values for the Zn extraction contents, the maximum extraction was obtained using the sequestering reagent at site 4 (soil from urban area). This is probably a result of the Cd-Pb-Zn enrichments in the urban agricultural land. Similar results were obtained for Fe extraction with H_2_O and with DTPA–CaCl_2_–TEA, where the efficiency was not significant (<1%). The acid reagent (0.1 mol L^−1^ HCl) is a very efficient extractability agent in the case of Ba, Ca, K, Mg, Mn and P. Therefore, the nutritive elements are mostly extracted at lower pH values with no significant variations between the sampling locations (Table 2, Figure 1).

#### Multivariate component statistics

**Figure 2:**
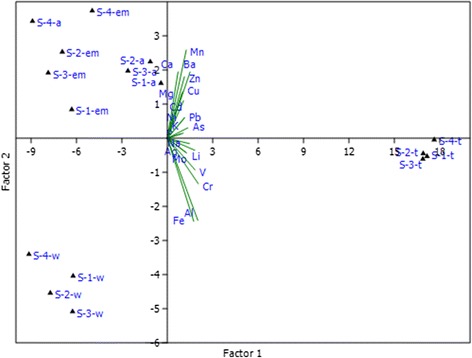
**Biplot for PCA-soil model for dependence of elements contents from different sampling locations, and different soil extraction solutions. w** - extraction with distillated water; **em** - extraction of elements in soil with DTPA–CaCl_2_–TEA; **a** - extraction with 0.1 HCl; **t** - total dissolved elements; **1**-site 1, Cu-mine environ; **2-**site 2, Former Fe-mine; **3**-site 3, reference area; **4**-site 4, urban area.

The obtained data for elements contents were processed using multivariate component statistics. In Figure 2 is presented bi-plot of principle component analysis for the metals contents in soil, their extractability power and dependence on sampling location. Two principle components (Factor 1 and Factor 2) were assumed with a total variation of 86%. The two components can separate well the anthropogenic (caused by mining activities) and lithogenic elements one from another. The anthropogenic elements (As, Cd, Cu, Pb, Zn) including Ca, Mg, Ba and Mn as naturally enriched, are included in factor 2, which positively correlates with DTPA-TEA-CaCl_2_ and HCl as extraction agents. Higher contents of these metals are characteristic for the soil samples from site 1 and 2, as previously reported [18]. These elements are grouped on the positive side of the PCA-plot (Figure 2). On the other hand, the typical lithogenic elements as Al, Ag, Cr, Fe, Mo, Li, V, and P-K as naturally enriched, are included in factor 1, in relation to which positive scores were established for total contents despite the water extractability (Figure 2). It can be summarized that both anthropogenic and lithogenic elements are not extracted in water solution, which means that their higher contents will be very hardly reduced by raining water or by watering the agricultural land. The potential availability of hazardous metals in urban and mining areas is recommended to be examined using DTPA-TEA-CaCl_2_ (for urban) and HCl (for Cu-mines areas).

### Plant species analysis

#### Characterization of metals contents-Basic descriptive statistics

Range and median values were used for presenting the data on elements contents in the plant species (Table 3). The presented values were calculated for the air-dried samples, while the moisture fraction ranges from 62.5-93%. The elements contents were analyzed separately in the edible vegetable parts (shoot) and the root. The essential macro elements (Ca, K, Mg and P) vary in their contents compared shoot to root. For example, potassium contents are enriched in *S. oleracea* (~3%). Maximum value for calcium content was obtained in the *U. dioica* shoot (2.1%) compared to the *R. acetosa* and *S. oleracea* (0.37 and 0.4%, respectively). The phosphorus content shows very low variability between shoot/root and between the analyzed vegetable species.

**Table 3:** **Basic statistics for elements contents in shoots and roots of plant species (contents are given in mg kg** ^**−1**^ **of dried matter)**

**Element**	***Rumex acetosa***				***Urtica dioica***				***Spinacia oleracea***			
	**Shoot**		**Root**		**Shoot**		**Root**		**Shoot**		**Root**	
	**Range**	**Med**	**Range**	**Med**	**Range**	**Med**	**Range**	**Med**	**Range**	**Med**	**Range**	**Med**
**Ag**	0.01-0.47	0.15	0.005-0.11	0.037	0.011-0.35	0.091	0.029-0.17	0.058	0.01-0.16	0.08	0.01-1.17	0.04
**Al**	18.8-293	55.8	174-406	328	76.0-386	223	214-1396	494	93.3-259	172	163-466	308
**As**	<0.25-0.94	<0.25	<0.25-1.18	0.76	<0.25-0.90	<0.25	0.53-0.94	0.89	<0.25-0.76	0.44	<0.25-0.40	<0.25
**Ba**	1.38-11.0	3.49	8.36-14.2	10.7	1.80-23.4	13.2	3.47-21.9	10.6	2.21-11.4	4.40	5.37-19.1	9.90
**Ca**	2405-5251	3777	4073-13523	5526	17689-26850	21763	4188-8798	4773	3252-9099	4049	2283-3863	2819
**Cd**	0.03-0.07	0.039	0.05-0.09	0.053	0.01-0.03	0.01	0.02-0.11	0.08	0.06-0.26	0.13	0.09-0.34	0.17
**Cr**	0.13-0.60	0.22	0.41-0.74	0.66	0.22-0.78	0.40	0.73-2.29	1.01	0.27-0.88	0.49	0.38-1.63	0.83
**Cu**	6.54-11.4	8.07	6.33-23.6	12.9	10.6-32.4	15.3	7.1-24.0	10.0	6.41-9.66	7.74	3.80-7.48	6.45
**Fe**	56.0-218	79.2	211-424	238	80.1-240	154	204-892	459	102-253	133	142-368	272
**K**	18945-30910	21751	5640-10711	8493	10292-17683	14800	9208-12105	10181	30197-38421	30809	19233-25846	21854
**Li**	0.06-0.31	0.081	0.15-0.22	0.18	0.040-0.37	0.17	0.08-0.88	0.31	0.089-0.35	0.13	0.14-0.30	0.20
**Mg**	1166-1783	1521	1051-1594	1192	866-1453	1237	393-1000	809	1680	1396	1058-1387	1237
**Mn**	11.3-30.3	17.7	6.89-16.5	11.5	16.5-77.7	23.8	18.1-48.4	31.4	12.0-70.1	23.3	10.8-33.9	19.9
**Mo**	0.33-1.24	0.76	0.27-1.65	0.57	0.54-2.05	1.26	0.03-0.50	0.09	0.089-0.84	0.51	0.05-0.37	0.15
**Na**	60.4-99.0	77.8	76.7-354	214	40.0-88.2	53.7	42.5-176	125	31.0-837	82.1	64.3-861	314
**Ni**	0.51-1.93	0.77	0.49-3.10	1.55	1.45-1.67	1.51	0.89-3.78	3.32	0.64-3.09	1.45	0.85-3.06	1.84
**P**	1540-3995	1927	1041-1766	1418	2081-3275	2506	1906-2492	2225	1962-3305	2810	2833-4507	3198
**Pb**	0.56-0.96	0.70	0.32-1.83	0.65	0.40-0.98	0.65	0.86-1.69	1.24	0.59-0.97	0.68	0.45-0.83	0.76
**Sr**	2.02-9.39	4.16	25.1-33.1	30.9	15.0-92.8	51.3	10.8-40.1	25.0	6.88-20.5	14.2	19.2-22.4	21.3
**V**	0.03-0.45	0.091	2.04-4.11	2.91	0.10-0.57	0.32	1.27-3.65	1.93	0.15-0.55	0.32	0.49-1.27	0.95
**Zn**	18.6-29.3	23.3	15.0-31.8	23.7	12.9-36.2	15.4	12.4-167	20.9	39.7-66.5	56.8	26.2-32.3	28.6

The content of the essential trace elements (Cu, Fe, Mn, Mo and Zn) shows higher levels than usually found [16-18]. The essential need of the plant tissue for Cu is ~0.9 mg kg^−1^ [11]. However, the determined contents of the copper in the present study is enriched to 32 mg kg^−1^ in the *U. dioica* shoot (sample collected very close to the copper mine). The essential content of Fe for the plant tissue is 18 mg kg^−1^ [11]. The determined iron content varies between shoot/root accumulation and between the species. The highest Fe content was obtained for the *U. dioica* root (892 mg kg^−1^ at the reference site location), accompanied by higher shoot content (204 mg kg^−1^). Thus, it can be considered that the Fe-content does not depend on the anthropogenic enrichments in the mines environ. The manganese content, generally required as a cofactor in enzyme functions (2.3 mg kg^−1^), occurs in higher contents in the analyzed vegetables, without any significant variations (ranging from 6.9-77 mg kg^−1^). Maximum Mn values were obtained for the *U. dioica* and *S. oleracea* shoot. Molybdenum as an essential trace element required in the amount of 0.045 mg kg^−1^ as given by Gunduz et al. [4] does not occur in higher contents (ranging from 0.03-1.65 mg kg^−1^). Zinc, as one of the most characteristic essential trace elements, is pervasive and required for several enzymes such as carboxyl peptidase, liver alcohol dehydrogenase, and carbonic anhydrase with nominal needs of 11 mg kg^−1^ [11]. There was enriched accumulation of Zn in the vegetables, ranging from 12.4-167 mg kg^−1^. The maximum Zn content was obtained for the *U. dioica* root from site 2.

The other trace elements (As, Cd, Cr and Pb) in higher contents can produce toxic effects on the vegetables, or negative effects on humans consuming them as food. As regards arsenic, half of the values found were below the limit of detection, for all three vegetable species. Only in the urban area the arsenic content was found as potentially hazardous (0.21-0.34 mg kg^−1^), especially in the *U. dioica* root and the *R. acetosa* root and shoot. The maximum permitted values for Cd and Pb content in fresh vegetables are regulated by established standard rules in the Republic of Macedonia, considering 0.2 and 0.3 mg kg^−1^, respectively, as the highest permitted contents [29]. Higher values for Cd contents in the *U. dioica* and *R. acetosa* were obtained from the urban site location. The maximum value for Cd content was obtained from the *S. oleracea* root (0.34 mg kg^−1^, values for dried mass fraction) in the urban area. However, the Cd content in these species, including the moisture mass fraction, alleviates the situation, because the obtained value was below 0.3 mg kg^−1^. Lead was also analyzed as a potentially toxic metal, due to the copper anthropogenic enrichment in the investigated area, as previously considered [16,17]. The values for Pb contents range from 0.4 to 2 mg kg^−1^ for dried mass, while for the fresh matter (including the moisture fraction mass) they range 0.05-0.41 mg kg^−1^, pointing to partial contamination (maximum permitted contents - 0.3 mg kg^−1^ in fresh matter). Hazardous Pb contents were obtained from *U. dioica* and *R. acetosa.* The maximum value for Pb content (0.41 mg kg^−1^) was obtained from the former Fe mine environ (site 2) in the *R. acetosa* root, while as regards the shoot and root of the *U. dioica* shoot and root, higher values were obtained from the Cu-mine environ and from the urban area.

Chromium belongs to the group of essential micro-nutrients. The Cr contents ranges from 0.13 mg kg^−1^ to 2.29 mg kg^−1^. However, the inorganic chromium absorption is quite low i.e. 1-2% of the available content in the soil. The Cu contents were in the range of 6.33-32 mg kg^−1^ (for dried vegetables). Considering the Cu-mine at site-1, the maximum value for Cu-content was obtained from the *U. dioica* root (8.65 mg kg^−1^ for fresh plant tissue). There is no established permitted value for Cu, but compared with the Cu content in the *U. dioica* from the reference area (7.45 mg kg^−1^), there is a slight enrichment. Significant enrichments were established for the Fe content in plant tissues for the *R. acetosa* < *S. oleracea* < *U. dioica* roots. However, this occurrence was expected, especially regarding the location (2), where a former Fe-mine is located. Soil analysis has shown Fe contents ~3%. For the rest of the elements there is no significant variation in the metals contents between the plant species and the sampling locations.

#### Metals correlations and associations in vegetables/herbs

Correlations between the elements contents in the investigated vegetables were established using bivariate statistics. The significant correlations are marked in bold in Table 4. The correlations of Fe-Al (r = 0.95), Fe-Cr (r = 0.91), Cr-Al (0.92) were selected as significant relations for the lithoogenic elements accumulation. The lithogenic impact of the region is related with the Eocene flysh and molase as well as the impact of oldest formation: Pleistocene sediments and Proterozoic gneisses, as given by Balabanova et al. [18] and Stafilov et al. [19]. The correlation Cu-Cd, relies on the anthropogenic influence of the copper mine as previously was found using moss and lichen plant species as sampling media [16,17].

**Table 4:** **Matrix of correlation coefficients**

Ag	1.00																				
Al	−0.11	1.00																			
As	0.14	0.30	1.00																		
Ba	0.18	0.74**	0.25	1.00																	
Ca	0.06	0.20	0.17	0.27	1.00																
Cd	−0.08	0.26	0.11	0.08	−0.50*	1.00															
Cr	−0.16	0.92**	0.33	0.58**	0.06	0.48	1.00														
Cu	0.32	0.03	0.32	0.14	0.53**	−0.66**	−0.16	1.00													
Fe	−0.10	0.95**	0.34	0.66**	0.07	0.33	0.91**	0.10	1.00												
K	0.08	−0.42*	−0.44*	−0.36	−0.39	0.37	−0.32	−0.47*	−0.45*	1.00											
Li	0.08	0.86**	0.36	0.72**	0.16	0.30	0.83**	0.06	0.81**	−0.19	1.00										
Mg	−0.01	−0.17	−0.20	0.01	0.02	0.16	−0.18	−0.16	−0.21	0.46*	0.11	1.00									
Mn	0.06	0.27	0.03	−0.01	0.31	0.13	0.32	0.04	0.24	0.24	0.25	0.09	1.00								
Mo	0.09	−0.47*	0.00	−0.39	0.44*	−0.51*	−0.54**	0.55**	−0.42*	−0.02	−0.34	0.26	0.07	1.00							
Na	−0.11	0.28	0.19	0.12	0.02	0.40	0.37	−0.31	0.28	−0.09	0.41*	0.24	−0.05	−0.09	1.00						
Ni	0.07	0.47*	0.17	0.33	0.10	0.38	0.52**	0.01	0.56**	−0.15	0.33	−0.22	0.42*	−0.29	−0.05	1.00					
P	−0.10	−0.22	−0.31	−0.30	−0.38	0.18	−0.06	−0.36	−0.20	0.54**	−0.15	−0.14	0.22	−0.17	−0.09	0.10	1.00				
Pb	0.05	0.62**	0.36	0.38	−0.06	0.29	0.64**	0.01	0.64**	−0.27	0.53**	−0.03	0.23	−0.37	0.00	0.59**	−0.07	1.00			
Sr	0.09	0.64**	0.30	0.81**	0.61**	−0.12	0.49*	0.33	0.53**	−0.49	0.57**	−0.11	0.02	−0.09	0.21	0.28	−0.26	0.25	1.00		
V	−0.20	0.83**	0.37	0.59**	0.07	0.28	0.78**	0.08	0.88**	−0.66**	0.62**	−0.28	−0.09	−0.38	0.33	0.43*	−0.43*	0.50*	0.52**	1.00	
Zn	−0.03	−0.28	−0.04	−0.54**	−0.31	0.56**	−0.06	−0.29	−0.16	0.40	−0.32	−0.03	0.09	−0.02	−0.03	0.26	0.22	0.08	−0.45*	−0.15	1.00
	Ag	Al	As	Ba	Ca	Cd	Cr	Cu	Fe	K	Li	Mg	Mn	Mo	Na	Ni	P	Pb	Sr	V	Zn

**Correlations (Box-Cox transformed values), the marked correlations are significant at p <0.01000. N = 24.

*Correlations (Box-Cox transformed values), the marked correlations are significant at p < 0.05000. N = 24.

The multivariate data processing method was also applied to reduce and optimize the numerous data matrix. Factor analysis was used to minimize the elements distribution. Using the *engine-values screening* in data correlation (>1.00), the distribution was reduced to two factors (associations of elements). The matrix for factor loadings is given in Table 5. Elements associations depend on their geogenic and anthropogenic origins. Two significant components were identified: F1 (Ag-Al-As-Ba-Cr-Fe-Li-Mg-Mn-Ni-V) and F2 (Ca-Cd-Cu-K-P-Mo-Na-Pb-Zn), as given in Table 5. The correlations of elements contents in different plant parts (shoot and root) were analyzed. The factor patterns were also examines for possible correlation with the elements’ factor associations (Table 6). *R. acetosa* significantly correlates with the F1 association, which means that this species shows affinity for geogenic elements. From the other side, *U. dioica* and *S. oleracea* differ in their factor correlations, primarily depending on the environmental conditions, which mean that a specific accumulation does not occur (Table 6). Considering the sampling locations, for site 1 and 2 most of the patterns loading have significance for Factor 1, while the vegetables and herbs sampled from the urban area (site 4) have significance for Factor 2. The samples collected from the reference area (site 3) differ between F1 and F2 (Table 6).

**Table 5:** **Factor loadings for elements contents (F > 0.50)**

	**F1**	**F2**	**Initial communality**	**Final communality**	**Specific variance**
Ag	**−0.076**	0.058	0.844	0.009	0.991
Al	**0.955**	0.090	0.990	0.919	0.081
As	**0.392**	−0.234	0.852	0.209	0.791
Ba	**0.683**	−0.309	0.990	0.562	0.438
Ca	0.119	**−0.688**	0.964	0.487	0.513
Cd	0.122	**0.787**	0.989	0.635	0.365
Cr	**0.882**	0.380	0.982	0.923	0.077
Cu	0.155	**−0.680**	0.917	0.486	0.514
Fe	**0.934**	0.121	0.988	0.887	0.113
K	−0.486	**0.507**	0.946	0.493	0.507
Li	**0.860**	0.070	0.986	0.745	0.255
Mg	**−0.223**	0.028	0.977	0.051	0.949
Mn	**0.127**	0.124	0.878	0.031	0.969
Mo	−0.336	**−0.514**	0.955	0.378	0.622
Na	0.071	**0.430**	0.954	0.190	0.810
Ni	**0.516**	0.293	0.906	0.352	0.648
P	−0.219	**0.400**	0.937	0.207	0.793
Pb	0.165	**0.662**	0.959	0.465	0.535
V	**0.700**	−0.043	0.883	0.492	0.508
Zn	−0.108	**0.323**	0.972	0.116	0.884

F1, F2 -factor loadings, values in bold correspond for each variable to the factor for which the squared cosine is the largest, (Box-Cox transformed values).

**Table 6:** **Factor loadings for vegetable species in relation to factors obtained for elements associations**

**Code**	**Plant species/part**	**F1**	**F2**
RA 1	*Rumex acetosa* shoot	**−1.120**	−0.171
	*Rumex acetosa* root	−0.398	**1.489**
UD 1	*Urtica dioica* shoot	−1.129	**0.945**
	*Urtica dioica* root	**1.226**	0.483
SO 1	*Spinacia oleracea* shoot	**−1.044**	−0.712
	*Spinacia oleracea* root	**−0.608**	−0.593
RA 2	*Rumex acetosa* shoot	**−1.170**	−0.100
	*Rumex acetosa* root	**0.864**	0.171
UD 2	*Urtica dioica* shoot	**−1.154**	0.392
	*Urtica dioica* root	0.427	**−0.799**
SO 2	*Spinacia oleracea* shoot	−0.255	**−0.834**
	*Spinacia oleracea* root	0.225	**−0.698**
RA 3	*Rumex acetosa* shoot	**−1.250**	−0.382
	*Rumex acetosa* root	**0.237**	0.290
UD 3	*Urtica dioica* shoot	0.024	**2.142**
	*Urtica dioica* root	**3.410**	0.230
SO 3	*Spinacia oleracea* shoot	**−0.410**	−0.765
	*Spinacia oleracea* root	0.434	**−0.798**
RA 4	*Rumex acetosa* shoot	−0.087	**−0.208**
	*Rumex acetosa* root	0.212	**1.039**
UD 4	*Urtica dioica*shoot	0.208	**1.969**
	*Urtica dioica* root	**0.426**	0.426
SO 4	*Spinacia oleracea* shoot	0.085	**−1.371**
	*Spinacia oleracea* root	0.848	**−2.145**

Values in bold correspond for each observation to the factor for which the squared cosine is the largest; RA-*Rumex acetosa*, UD-*Urtica dioica*, SO-*Spinacia oleracea*, Site 1- copper mine environ and Site 2 – Former Fe-mine environ; Site 3 – reference area, non-contaminated area; Site 4 – urban area.

**Figure 3:**
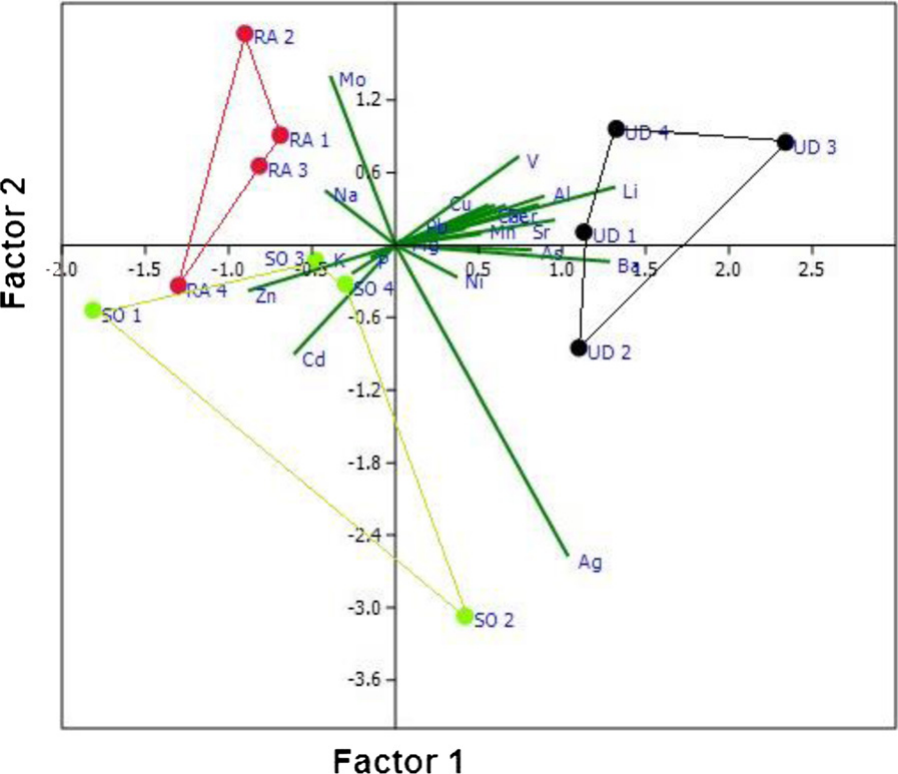
**Principle components of vegetable species and its elements contents.** RA *- Rumex acetosa;* UD *- Urtica dioica;* SO *- Spinacia oleracea;* Site 1 and 2 – polluted areas; Site 4 – urban area; Site 3 – control area, non-contaminated area.

Screen plot and PC loadings for PCA2 - plant model are presented in Figure 3. It is obvious from this model that the first two PCs explain only 64.4% of the variance. Graphical representation of the PCA2 - plant model is given on Figure 3. The two factors can well separate the three vegetables/herbs species in relation to their elements accumulation. The *R. acetosa* species occupies an area strongly correlated with higher contents of Na and Mo; there are certain natural phenomena that control the specific trend in their accumulation. The first quartile in the PCA graph confirms the specific trends in the correlation of Cd and Zn accumulation from sorrel in urban areas (Figure 3). *Spinacia oleracea* species are mainly correlated with higher contents of Zn and Cd, as given previously in Table 4, the significant correlations Cd-Zn (0.56). Common nettle (*U. dioica*) shows particular specificity for accumulation of certain elements. Some of the lithogenic elements such as Al, Cr, Li, Mn, Sr and V show specific correlation with the *U. dioica* accumulation. The samples of *S. oleracea* and *U. dioica* collected from the former Fe mine area (site 2) are characterized with specific accumulation of Ag, As, Ba and Ni. This certainly relies on the geology of the area and the specific ability of these certain species to accumulate the respective contents, despite their lower contents in the soil.

#### Bioavailability and translocation metals in R. acetosa, S. oleracea and U. dioica

TF, BCF and BAC values >1 had been used to evaluate the potential of plant species for phytoextraction and phytostabilization of metals in soil [7]. For the macro biogenic elements TF values were in the order of K > P > Mg > Na > Ca for *R. acetosa*. For *S. Oleracea* and *U. dioica* the following order applies Ca > K > Mg > P > Na (Table 7). Regarding the lithogenic elements there are no significant variations in the TF values among all three plants species: Al (0.11-0.92); Ba (0.15-1.87); Cr (0.21-0.87); Li (0.34-1.86); Ni (0.16-1.76) and V (0.01-0.45) as given in Table 7. For molybdenum higher TF values were obtained ranging from 0.75-9.47, with emphasis on *U. dioica*. For copper, as the main anthropogenic element in the investigated area, significant TF values were obtained: for *R. acetosa* ranging from 0.48-1.30; for *U. dioica* from 1.12-4.56; and for *S. oleracea* from 1.04-1.68 (Table 7). For Cd contents in *R. acetosa* and *S. oleracea* similar TF values were obtained for all four location sites. *Urtica dioica* varies in TF for Cd, with a significant value (1.83) obtained from the urban area. The translocation factor for Pb was higher in the *R. acetosa* as plant species (2.12), considering the location site as the most polluted from the Cu mine, where As, Cd, Pb and Zn were found in higher contents in soil. Considering this, the TF values for almost all of the analyzed elements are in range from 0.1-9.5. Higher root to shoot translocation of these metals indicated that these plants have vital characteristics to be used for phytoextraction of these metals as indicated by Ghosh and Singh [12]. According to Marschner [11] and Overesch et al. [13] *R. acetosa* has the highest translocation efficiency for Ni and Cr. In the present study *R. acetosa* had higher TF values for Cd, Cu, Ni and Pb. Thus, this species could have the potential to be used for phytoextraction of the mentioned metals in potentially polluted areas. *Urtica dioica* showed potential to be used for phytoextraction only for Cu but not much specific potential for Pb, as previously investigated by Grubor [30].

**Table 7:** **Estimation of analyzed elements from plant species**

**Elements**		**Ag**	**Al**	**As**	**Ba**	**Ca**	**Cd**	**Cr**	**Cu**	**Fe**	**K**	**Li**	**Mg**	**Mn**	**Mo**	**Na**	**Ni**	**P**	**Pb**	**V**	**Zn**
Translocation factor-TF																					
*R. acetosa*	1	2.05	0.11	0.92	0.15	0.18	0.61	0.43	0.48	0.26	**2.85**	0.66	0.91	0.88	0.75	0.21	0.57	**3.84**	**2.12**	0.01	0.92
	2	0.34	0.17	0.49	0.21	0.63	0.36	0.35	0.50	0.23	**2.01**	0.29	**1.07**	**1.43**	**1.39**	0.27	0.16	**1.04**	0.39	0.05	0.85
	3	6.20	0.11	**1.05**	0.43	**1.05**	0.90	0.21	**1.03**	0.23	**2.89**	0.34	**1.21**	**1.72**	**1.13**	0.75	0.41	**1.18**	0.41	0.01	**1.38**
	4	1.07	0.92	0.21	0.77	0.90	**1.35**	0.87	0.75	1.03	**3.90**	**1.86**	**1.70**	**2.98**	**1.23**	0.79	3.94	**1.38**	**1.39**	0.16	**1.08**
*U.dioica*	1	7.00	0.22	0.26	0.61	**3.49**	0.07	0.21	**1.18**	0.17	**1.46**	0.26	**1.68**	**1.60**	**4.06**	0.56	0.49	**1.12**	0.45	0.05	0.58
	2	0.05	0.35	0.58	0.52	**4.22**	0.05	0.31	**1.12**	0.39	**1.66**	0.51	**1.84**	**1.54**	**9.47**	0.94	0.44	**1.72**	0.26	0.07	0.21
	3	3.66	0.23	0.29	**1.07**	**2.93**	0.05	0.25	**4.56**	0.23	**1.34**	0.42	0.76	0.38	**1.30**	0.32	0.46	**1.03**	0.50	0.14	0.77
	4	1.16	0.81	0.98	**1.87**	**6.03**	**1.83**	0.81	**1.14**	0.80	**1.06**	0.97	0.83	0.98	**5.01**	0.56	**1.76**	0.91	**1.13**	0.45	**1.26**
*S.oleracea*	1	2.33	0.57	**1.01**	0.41	**1.56**	0.68	0.71	**1.35**	0.72	**1.59**	0.73	**1.09**	**1.11**	**1.52**	0.25	0.75	**1.16**	**1.31**	0.31	**2.49**
	2	0.03	0.38	**2.56**	0.60	**1.50**	**1.05**	0.46	**1.04**	0.40	**1.32**	0.53	**1.42**	**1.13**	**1.57**	0.59	**1.28**	0.98	**1.16**	0.27	**1.33**
	3	0.32	0.55	0.62	0.28	**1.23**	0.59	0.67	**1.29**	0.69	**1.17**	0.53	0.86	**1.20**	**1.93**	0.24	**1.11**	0.80	**1.09**	0.44	**1.76**
	4	1.61	0.72	**3.04**	0.67	**2.36**	0.75	0.54	**1.68**	0.59	**1.89**	**1.50**	**1.30**	**2.06**	**3.71**	0.97	0.48	0.44	**1.07**	0.39	**2.05**
Bioaccumulation factor - BAF																					
*R. acetosa*	1	0.49	<0.01	0.03	<0.01	0.11	0.03	<0.01	0.11	<0.01	*0.91*	0.01	0.11	0.02	0.31	0.01	0.05	**2.88**	0.02	<0.01	0.37
	2	0.01	<0.01	0.01	<0.01	0.16	0.03	0.01	0.11	<0.01	*0.96*	0.01	0.26	0.04	0.37	0.01	0.02	**1.97**	0.02	<0.01	0.37
	3	0.13	<0.01	<0.01	0.02	0.19	0.05	0.00	0.19	<0.01	**1.91**	0.01	0.15	0.02	0.12	0.01	0.01	**2.03**	<0.01	<0.01	0.28
	4	0.43	<0.01	<0.01	0.03	0.15	0.11	0.01	0.12	0.01	**1.08**	0.03	0.21	0.05	0.13	0.01	0.06	*0.71*	0.01	0.01	0.10
*U.dioica*	1	*0.75*	<0.01	<0.01	0.02	*0.78*	<0.01	<0.01	0.28	<0.01	*0.75*	0.01	0.11	0.15	*0.51*	0.01	0.07	**2.01**	0.02	<0.01	0.16
	2	0.01	<0.01	<0.01	0.00	*0.87*	<0.01	0.01	0.16	<0.01	*0.77*	0.01	0.13	0.05	0.38	<0.01	0.07	**3.52**	0.01	<0.01	0.49
	3	0.23	0.01	<0.01	0.07	**1.17**	<0.01	0.01	*0.94*	0.01	**1.91**	0.05	0.16	0.03	0.20	0.01	0.04	**2.24**	0.02	0.01	0.21
	4	0.07	0.01	0.02	0.05	*0.76*	0.06	0.01	0.15	0.01	**1.08**	0.03	0.15	0.03	*0.52*	0.01	0.05	*0.95*	0.01	0.01	0.10
*S.oleracea*	1	0.26	<0.01	0.01	0.01	0.21	0.06	<0.01	0.08	<0.01	**1.29**	0.01	0.12	0.02	0.14	0.01	0.03	**2.39**	0.02	<0.01	*0.82*
	2	0.04	<0.01	0.03	0.03	0.17	0.17	0.01	0.10	<0.01	**1.56**	0.02	0.23	0.03	0.03	<0.01	0.13	**2.99**	0.03	<0.01	*0.54*
	3	0.01	<0.01	0.01	0.01	0.12	0.12	0.01	0.28	0.01	**1.87**	0.02	0.13	0.05	0.17	<0.01	0.04	**2.85**	0.02	0.01	*0.66*
	4	0.15	<0.01	0.02	0.01	0.11	0.43	0.02	0.18	<0.01	**1.89**	0.04	0.19	0.12	0.34	0.11	0.04	*0.90*	0.01	0.01	0.36
Bioconcentrating factor (BCF)																					
*R.acetosa*	0.24	<0.01	0.04	0.03	*0.60*	0.05	0.01	0.24	0.01	0.32	0.02	0.12	0.02	0.41	0.03	0.09	*0.75*	0.01	0.02	*0.40*	0.24
	0.04	0.01	0.02	0.02	0.25	0.09	0.02	0.21	0.01	0.48	0.04	0.24	0.03	0.26	0.04	0.13	**1.90**	0.04	0.04	*0.42*	0.04
	0.01	<0.01	0.01	0.04	0.19	0.06	0.01	0.18	0.01	*0.66*	0.02	0.12	0.01	0.11	0.02	0.04	**1.73**	0.02	0.05	0.20	0.01
	0.04	<0.01	0.02	0.03	0.17	0.09	*0.42*	0.15	0.01	0.28	0.02	0.12	0.02	0.11	0.01	0.01	*0.51*	0.01	0.04	0.09	0.04
*U.dioica*	0.11	0.01	0.04	0.03	0.22	0.07	0.02	0.24	0.01	*0.51*	0.04	0.07	0.09	0.13	0.01	0.18	**1.80**	0.04	0.03	0.28	0.11
	0.23	<0.01	0.02	0.01	0.20	0.11	0.02	0.15	0.01	0.46	0.01	0.06	0.03	0.04	<0.01	0.16	**2.05**	0.03	0.02	**2.30**	0.23
	0.06	0.02	0.05	0.06	0.40	0.11	0.04	0.21	0.03	*0.66*	0.11	0.11	0.08	0.02	0.02	0.08	**2.17**	0.05	0.04	0.27	0.06
	0.06	0.01	0.02	0.02	0.13	0.03	0.02	0.13	0.01	0.48	0.03	0.10	0.03	0.01	0.02	0.03	**1.05**	0.01	0.02	0.07	0.06
*S.oleracea*	0.11	<0.01	0.01	0.02	0.13	0.09	0.01	0.17	0.00	*0.81*	0.02	0.11	0.02	0.09	0.06	0.04	**2.06**	0.02	0.01	*0.33*	0.11
	*0.54*	<0.01	0.01	0.05	0.11	0.16	0.02	0.10	0.01	**1.18**	0.03	0.16	0.03	0.02	0.01	0.10	**3.04**	0.02	0.01	*0.41*	*0.54*
	0.07	0.01	0.02	0.03	0.12	0.21	0.02	0.22	0.01	**1.60**	0.04	0.15	0.04	0.09	0.02	0.03	**3.56**	0.02	0.02	*0.37*	0.07
	<0.01	<0.01	0.01	0.02	0.11	*0.57*	0.03	0.15	0.01	**1.00**	0.02	0.15	0.06	0.01	0.11	0.09	**2.06**	0.01	0.01	0.18	<0.01

Values >1 are bolded, indicating significant efficiency; values in range of 0.50-0.99 for BAF and BCF are italic, indicating potential efficiency; 1-site 1, Cu mine environ, 2-site 2, former Fe mine environ, 3 – site 3 reference area, 4-site 4, urban area.

The bioaccumulation factor (BAF) of the studied plant species was in the lower range (<1) for almost all of the analyzed metals (Table 7). The macro-biogenic elements for all three species had significant BAF values; for Ca in the range of 0.11-1.17, for K: 0.75-1.91, for Mg: 0.11-0.33, and for P: 0.71-2.99). The bioaccumulation factors for Cu and Zn were in the range from 0.10-0.94 and 0.10-0.84, respectively. For the other potentially hazardous metals such as As, Cd and Pb, the bioacummulation ratios ranged <0.01-0.06; <0.01-0.43 and 0.01-0.03, respectively. The maximum BAF_Cd_ and BAF_As_ for all plant species were obtained from the urban area (site 4). While the metal contents ratio root/soil for Cu, Ni, and Pb varies, no specific variations related to the sampling location were established.

Root/soil ratio (bioconcentration factor -BCF) was used for qualification of plant species for phytostabilization of contaminated soils. Regarding the bioconcentration ratios, for the majority of the elements values smaller than 1 were obtained (Table 7). The BCF values in the range of 0.1-0.50 (as potential phytostabilization) and values from 0.5-1.0 (as partial phytostabilization) should not be neglected. The BCF values for Cu were in the range from 0.10-0.24, for Zn in the range from 0.07-2.30 (the max. value was obtained for *U. dioica* at location 2). For Cd a BCF value of 0.57 was obtained for *S. oleracea* and for Cr a BCF value of 0.42 for *R. acetosa* in the urban area (Table 7). Despite the heavy metals, S. *oleracea* shows higher BCF values for K and P (1.60 and 3.56, respectively), which means that from soils that are fertilized without control, higher contents of P and K can be phytostabilized.

## Conclusions

This study shows that even in lower contents in partially contaminated soil As, Cd, Cu, Ni and Pb are very extractible and available from cultivated plants. Many authors suggested that common nettle (*U. dioica*) and spinach (*S. oleracea*) are very suitable phytoextraction plants in very polluted areas, but this study revealed that common nettle (*U. dioica*), spinach (*S. oleracea*) and sorrel (*R. acetosa*) are mostly efficient for bioaccumulation of Cd and Pb in potentially polluted areas. None of the species was specified as a hyper accumulator; nevertheless all three species show potential for phytoextraction and phytostabilization of Cd, Cu, Pb and Zn. In addition, further studies are required to determine the growth performance, biomass production and metal accumulation of these species in metal contaminated soils for their better management, conservation and assurance of better food quality when growing on urban, industrial and agricultural land near mines.
